# Mitral Valve Annulus Dimensions Assessment with Three-Dimensional Echocardiography Versus Computed Tomography: Implications for Transcatheter Interventions

**DOI:** 10.3390/jcm10040649

**Published:** 2021-02-08

**Authors:** Kensuke Hirasawa, N. Mai Vo, Tea Gegenava, Stephan Milhorini Pio, Suzanne E. van Wijngaarden, Nina Ajmone Marsan, Jeroen J. Bax, Victoria Delgado

**Affiliations:** Department of Cardiology, Heart Lung Centre, Leiden University Medical Center, 2333 ZA Leiden, The Netherlands; k.hirasawa@lumc.nl (K.H.); n.m.vo@lumc.nl (N.M.V.); t.gegenava@lumc.nl (T.G.); stephanpio@yahoo.com.br (S.M.P.); s.vanwijngaarden@gmail.com (S.E.v.W.); n.ajmone@lumc.nl (N.A.M.); j.j.bax@lumc.nl (J.J.B.)

**Keywords:** computed tomography, transesophageal echocardiography, mitral valve

## Abstract

The aim of this study is to evaluate the agreement between three-dimensional (3D) transesophageal echocardiography (TEE) and multidetector computed tomography (MDCT) for assessing mitral annular (MA) dimensions. A total of 105 patients (79 ± 9 years old, 52% male) who underwent clinically indicated 3D TEE and MDCT feasible for MA geometrical assessment were included. Using dedicated semi-automated postprocessing software, MA geometry, including mitral annular area (MAA), perimeter, septal-lateral (SL) diameter, and inter-trigonal (TT) diameter, was evaluated using 3D TEE and MDCT. Compared to 3D TEE, MAA, perimeter, and SL distance measured on MDCT data were larger (9.9 ± 3.0 vs. 9.3 ± 3.1 cm^2^ for MAA; 115 ± 18 vs. 108 ± 18 mm for perimeter; and 35 ± 5 vs. 32 ± 5 cm for SL distance, all *p* < 0.001). By contrast, the TT distance was comparable between MDCT and 3D TEE (26 ± 4 vs. 26 ± 4 cm, *p* = 0.258). The correlations of all the MA dimensions were good to excellent between the two modalities (R = 0.911 for MAA, 0.890 for perimeter, 0.739 for TT distance, and 0.857 for SL distance, respectively, all *p* < 0.001). This study showed good agreement between 3D TEE- and MDCT-derived MA measurements although MDCT systematically provided larger MAA, perimeter, and SL distance compared with 3D TEE.

## 1. Introduction

Based on the success of the transcatheter aortic valve implantation [1,2], transcatheter mitral valve (MV) implantation techniques have been developed [3,4]. The mitral valve annulus (MA) has a three-dimensional (3D) saddle-shape geometry with the highest point at the level of the mitro-aortic continuity and the deepest points at the level of the commissures of the mitral valve (MV) that cannot be appreciated using a two-dimensional imaging technique [5]. Therefore, the measurement of the MA to select the most appropriate size of the transcatheter valve to be implanted requires an imaging technique that is 3D. While echocardiography is the mainstay imaging technique to assess the MV function, multidetector row computed tomography (MDCT) is the key imaging technique to assess the dimensions of the MA in patients who are candidates for transcatheter MV implantation [6,7]. However, comparison between semi-automated software to assess the MA dimensions and geometry based on 3D transesophageal echocardiographic (TEE) data and MDCT data have not been performed. In this study, we aimed to evaluate the agreement between these two imaging modalities for assessing MA geometry using specific semi-automated software.

## 2. Materials and Methods

A total of 105 patients who underwent clinically indicated 3D TEE and MDCT suitable for MA assessment were included in this retrospective study. Demographics, cardiovascular risk factors, heart failure symptoms, and medications were collected from the departmental medical record system (EPD Vision; Leiden University Medical Center, Leiden, The Netherlands). The agreement between MA measurements conducted using MDCT and 3D TEE was evaluated. The study was conducted according to the guidelines of the Declaration of Helsinki, and the institutional ethical committee approved this retrospective analysis of clinically collected data (project identification code: CME10/024/SH, date of approval: 1 March 2010, institutional ethics committee: Leiden University Medical Center). The need for written informed consent was waived due to the retrospective design.

Comprehensive transthoracic echocardiography was performed in all patients using commercially available ultrasound systems (Vivid E9 or E95, GE Vingmed Ultrasound, GE Vingmed Ultrasound, Horten, Norway). All images were obtained according to current recommendations and digitally stored for offline analysis [8,9]. Left ventricular ejection fraction (LVEF) was calculated according to the Simpson’s biplane method [9]. Maximal left atrial volume was measured from the apical 4- and 2-chamber views as recommended and indexed to body surface area [9]. Severity of MV dysfunction (mitral stenosis or mitral regurgitation) was assessed according to the current guidelines [10,11].

**Figure 1:**
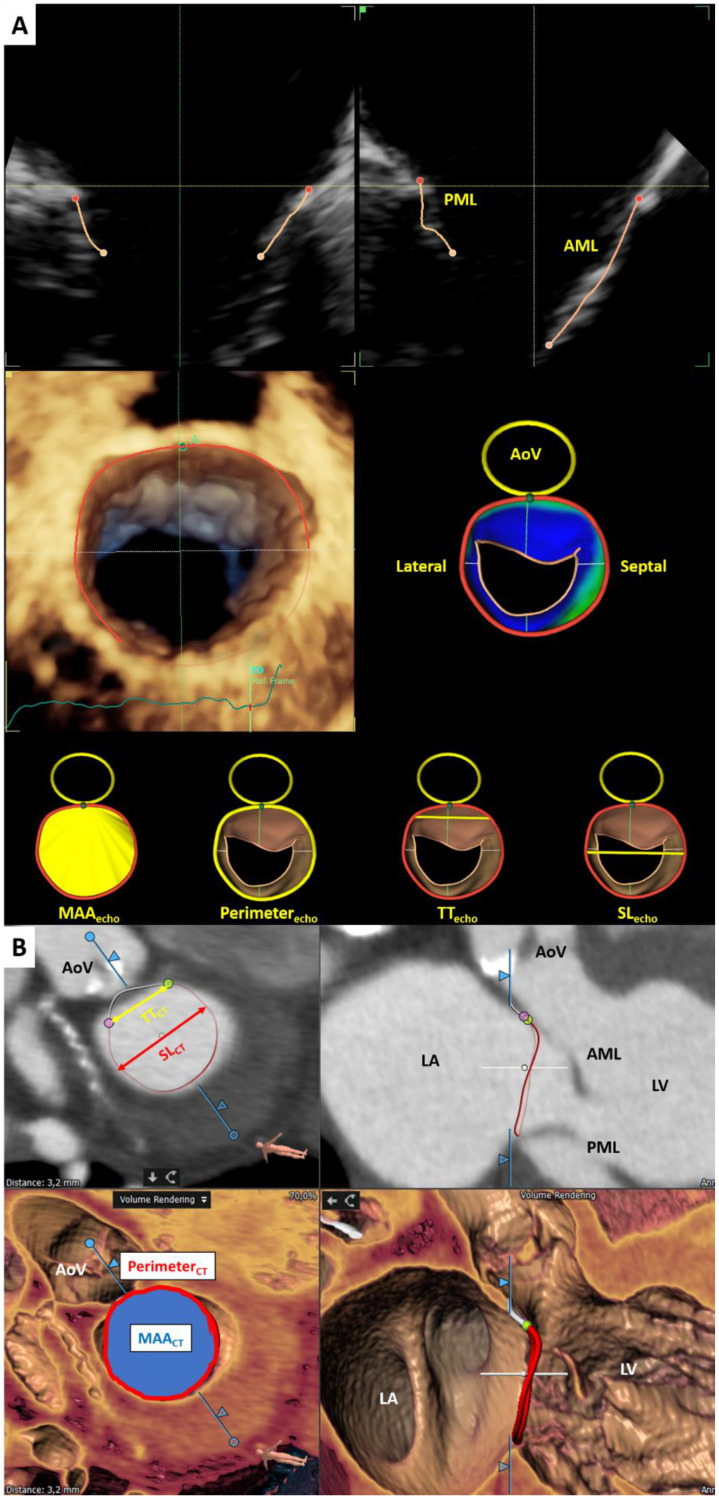
Assessment of mitral annular geometry using (**A**) 3D TEE and (**B**) MDCT. AoV = aortic valve; AML = anterior mitral leaflet; LA = left atrium; LV = left ventricle; MAA = mitral annular area; PML = posterior mitral leaflet; SL = septal-lateral distance; TT = inter-trigonal distance.

TEE was performed using the E95 ultrasound system equipped with a 4D transesophageal matrix array probe (6VT-D ultrasound transducer, GE Vingmed Ultrasound, Horten, Norway). A 3D full volume dataset of the mitral valve was acquired during sedation or general anesthesia. To optimize the temporal resolution, multi-beat acquisition was used during breath-holding whenever possible. The 3D full volume dataset of the mitral valve was digitally stored (Imagevault, GE, Horten, Norway) and analyzed using a dedicated workstation (EchoPAC Version 204, GE Medical Systems, Horten, Norway). The MA dimensions were measured in mid-diastole with novel semi-automated software (4D Auto MVQ, GE Healthcare, Horten, Norway) as previously described [12]. In detail, after selecting the mid-diastolic frame, multiplanar reconstruction planes were manually aligned to obtain the long-axis and inter-commissural views of the mitral valve. Subsequently, the anterior, posterior, posteromedial, and anterolateral coordinates of the MA as well as the coaptation point of the mitral valve leaflets and the hinge point of the right coronary cusp at the aortic annulus were defined. The software automatically defined the MA contours, which can be manually adjusted if needed. After final approval of the 3D reconstruction, the MA area, perimeter, inter-trigonal (TT) distance, and septal-lateral (SL) distance were automatically measured (Figure 1A).

MDCT scans were performed with a helical 64-detector (Aquilion64, Toshiba Medical Systems, Otawara, Japan) or a volumetric 320-detector row CT scanner (AquilionOne, Toshiba Medical Systems, Tochigi-ken, Japan) using a dedicated cardiac CT protocol, as previously described [13,14]. The MDCT data were analyzed using 3mensio software (version 10.0, Pie Medical Imaging, Bilthoven, The Netherlands) with dedicated application for mitral valve geometrical assessment (3mensio Valves workstation, version 10.0, Pie Medical Imaging, Bilthoven, The Netherlands). After selecting the mid-diastolic phase (70–80% of the cardiac cycle), the MA was automatically traced by manually defining 16 points around the axis perpendicular to the MA plane. Subsequently, the anterolateral and posteromedial trigone were identified as the points where the left- and non-coronary sinus meet the MA, respectively. Finally, the MA area (MAA), perimeter, TT distance, and SL distance were calculated automatically (Figure 1B).

Continuous variables are reported as mean ± standard deviation if normally distributed or median with interquartile range if not normally distributed. Categorical variables are reported as frequencies and percentages. The statistical significance of the mean difference between MA measurements derived from 3D TEE and MDCT was assessed using one-sample t-test. Subsequently, the limits of agreement and the mean bias between MDCT and 3D TEE measurements were plotted using Bland–Altman analysis. The correlation of MA parameters measured by MDCT and 3D TEE was evaluated using Spearman correlation analysis with correlation coefficient (R). *p* < 0.05 was regarded as statistically significant. All statistical analyses were performed using SPSS software (SPSS, Inc., Chicago, IL, USA).

## 3. Results

### 3.1. Patients Characteristics

A total of 105 patients (mean age of 79 ± 9 years, 52% male) were included. Patient baseline characteristics are shown in Table 1. The majority of the patients (*n* = 96, 91%) had severe aortic stenosis and underwent MDCT prior to transcatheter aortic valve implantation and 3D TEE was performed during the procedure. Nine patients had severe primary mitral regurgitation and underwent MDCT and 3D TEE prior to robotic mitral valve repair. Atrial fibrillation was observed in 18 patients (17%). The majority of patients (68%) presented with New York Heart Association (NYHA) class III/IV heart failure symptoms. Median left ventricular ejection fraction and left atrial volume were 56 (43–64)% and 46 (35–55) mL/m^2^, respectively.

### 3.2. MA Geometry Measured by MDCT and 3D TEE

**Figure 2:**
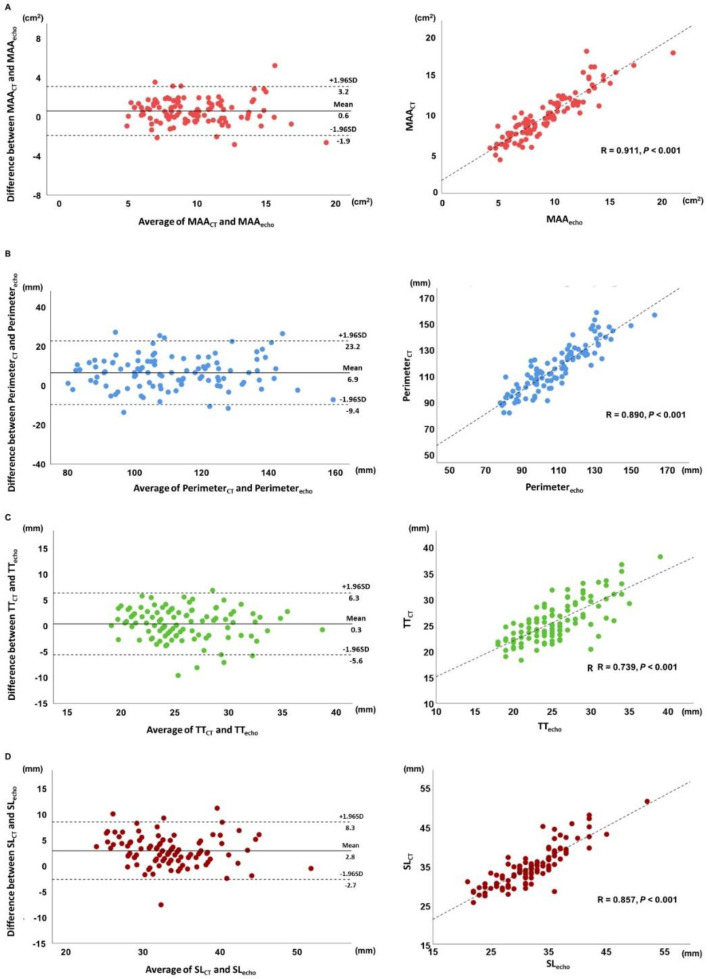
Bland–Altman and scatter plots for MDCT and 3D TEE measurements of MA geometry: (**A**) mitral annular area, (**B**) perimeter, (**C**) inter-trigonal distance, (**D**) septal-lateral difference. CT = computed tomography; MAA = mitral annular area; SD = standard deviation; SL = septal-lateral distance; TT = inter-trigonal distance.

The MA dimensions measured by MDCT and 3D TEE are shown in Table 2. The times for the assessment using the software are approximately 5 to 10 min per patient for both 3D TEE and MDCT. Compared to 3D TEE, MAA, perimeter, and SL distance measured on MDCT data were larger (9.9 ± 3.0 vs. 9.3 ± 3.1 cm^2^ for MAA; 115 ±18 vs. 108 ± 18 mm for perimeter; and 35 ± 5 vs. 32 ± 5 cm for SL distance, all *p* < 0.001). By contrast, the TT distance was comparable between MDCT and 3D TEE (26 ± 4 vs. 26 ± 4 cm, *p* = 0.258). Figure 2 shows the Bland–Altman and scatter plots of MA dimensions to demonstrate the agreement and correlation between MDCT and 3D TEE derived measurements. Good to excellent correlations were observed across all the measurements of the MA (R = 0.911 for MAA, 0.890 for perimeter, 0.739 for TT distance, and 0.857 for SL distance, all *p* < 0.001).

## 4. Discussion

The main findings of this retrospective analysis are as follows: (i) good agreement of D-shaped MA dimensions was observed between MDCT and 3D TEE; (ii) MAA, perimeter, and SL distance measured by MDCT were significantly larger compared to those by 3D TEE, whereas the TT distance was comparable between the two modalities.

MA has a dynamic saddle shape, and the assessment, therefore, requires 3D imaging modalities that acquire data throughout the entire cardiac cycle [5]. Moreover, evaluation of D-shaped MA dimensions, which include MAA, perimeter, SL distance, and TT distance, is essential for device sizing in the context of transcatheter MV implantation [6]. Using dedicated software, MDCT provides accurate MA dimensions, which include MAA, perimeter, SL distance, and TT distance with excellent spatial resolution [15,16]. TEE has also frequently been used for MV geometrical assessment and good reproducibility of 3D TEE-derived MV measurements has been reported in previous work [12,17]. In the present study, we demonstrated good agreement of MA dimensions between MDCT and 3D TEE using automated quantification software. However, MA dimensions measured with MDCT were frequently larger compared to those measured with 3D TEE. The reasons underlying this measurement bias may relate to the better soft-tissue resolution of MDCT data as compared with 3D TEE, since the use of intravenous contrast (with MDCT) permits better delineation of the cardiac structures. In addition, the presence of calcifications does not affect the demarcation of cardiac structures with MDCT, whereas with 3D TEE calcifications, it may lead to important shadowing of the structures that lay behind. Whether these measurement biases lead to a different selection of prosthesis size would be important to determine. However, the present patient population did not undergo TMVR, and this issue needs further study.

To date, several transcatheter MV intervention devices have been developed and applied into clinical practice for patients with symptomatic severe MR. In TMVR and direct transcatheter mitral annuloplasty, accurate assessment of 3D MA geometry is becoming important for device size selection and procedural success without complications [18,19,20]. Various articles reported on the utility of 3D MDCT assessment for procedural planning of transcatheter MV intervention [7,21]. However, the need for contrast media for MDCT is sometimes problematic since many of the patients who are referred for transcatheter MV intervention have severely impaired renal function. Alternatively, the use of 3D TEE for MA assessment obviates the risk of unnecessary exposure to contrast media and radiation. Thus, 3D TEE evaluation of MV geometry could be used a first-line imaging technique, eventually followed by MDCT in patients who need additional assessment.

The current study has limitations related to its retrospective design and the relatively small number of patients with mitral regurgitation included. In addition, the study population was heterogeneous, and the majority of the patients that had severe aortic stenosis were treated with transcatheter aortic valve implantation and TMVR techniques were not used.

## 5. Conclusions

MDCT and 3D TEE measurements of MA dimensions using semi-automated software showed good agreement, although MDCT systematically provided larger MAA, perimeter, and SL distance compared to 3D TEE. Whether this measurement bias may lead to different size of the prosthesis to be implanted needs further evaluation in which patients treated with transcatheter mitral valve replacement are included.

## Figures and Tables

**Table 1:** Patients’ demographic and clinical characteristics.

Parameters	*n* = 105
Age, years	79 ± 9
Male, *n* (%)	55 (52)
Body surface area, m^2^	1.87 ± 0.21
Prior MI, *n* (%)	21 (20)
Hypertension, *n* (%)	65 (62)
Hypercholesterolemia, *n* (%)	53 (51)
Diabetes, *n* (%)	35 (33)
Atrial fibrillation, *n* (%)	17 (18)
NYHA class, *n* (%)	
I	7 (7)
II	27 (26)
III/IV	71 (68)
ACEi/ARB, *n* (%)	51 (49)
Beta-blockers, *n* (%)	62 (59)
Ca-channel blockers, *n* (%)	22 (21)
Statins, *n* (%)	67 (64)
Diuretics, *n* (%)	45 (43)
LVEF, %	56 (43,64)
LVEDV, ml	101 (76,138)
LVESV, ml	43 (28,71)
LAVI, ml/m^2^	46 (35,55)
Mitral regurgitation	
Moderate, *n* (%)	20 (19)
Severe, *n* (%)	11 (11)
Mitral stenosis	
Mild, *n* (%)	12 (11)
Moderate–severe, *n* (%)	7 (7)

Values are mean ± standard deviation, median [interquartile range], or *n* (%). ACEi = angiotensin-converting enzyme inhibitor; ARB = angiotensin II receptor blocker; LAVI = left atrial volume index; LVEDV = left ventricular end-diastolic volume; LVEF = left ventricular ejection fraction; LVESV = left ventricular end-systolic volume; MI = myocardial infarction; NYHA = New York Heart Association.

**Table 2:** Mitral annular dimensions measured by MDCT and 3D TEE.

MV Annular Dimensions	MDCT *n* = 105	3D TEE *n* = 105	Bias (95% CI)	*p* Value of Bias
MAA, cm^2^	9.9 ± 3.0	9.3 ± 3.1	0.6 (−1.9, 3.2)	<0.001
Perimeter, mm	115 ± 18	108 ± 18	6.9 (−9.4, 23.2)	<0.001
TT distance, mm	26 ± 4	26 ± 4	0.3 (−5.6, 6.3)	0.258
SL distance, mm	35 ± 5	32 ± 5	2.8 (−2.7, 8.3)	<0.001

Values are mean ± standard deviation. MAA = mitral annular area; MDCT = multidetector computed tomography; MV: mitral valve; SL = septal-lateral; 3D TEE = three-dimensional transesophageal echocardiography; TT = inter-trigonal.
